# Dosage Optimization for Letrozole Treatment in Clomiphene-Resistant Patients with Polycystic Ovary Syndrome: A Prospective Interventional Study

**DOI:** 10.1155/2012/758508

**Published:** 2012-01-16

**Authors:** Elham Rahmani, Shahnaz Ahmadi, Niloofar Motamed, Hesam_Oddin Maneshi

**Affiliations:** ^1^School of Medicine, Bushehr University of Medical Sciences, Moallem Street, Bushehr, Iran; ^2^Department of Obstetrics and Gynecology, Bushehr University of Medical Sciences, Moallem Street, Bushehr, Iran; ^3^Department of Community Medicine, Bushehr University of Medical Sciences, Moallem Street, Bushehr, Iran

## Abstract

*Objective*. Dose adjustment for induction of ovulation is one of the most important problem. *Methods*. In a prospective interventional study, 44 clomiphene-resistant infertile patients (113 cycles) were selected from the Abolfazl Infertility Clinic of Bushehr University of Medical Sciences. Letrozole was given orally in a dose of 2.5 mg, 5 mg, and 7.5 mg, respectively. If the patient displayed no response, the dosage was increased. *Results*. In this patients ovulation occurred in 50 cycles (44.24%), clinical pregnancy rate according to number of cycles was 23.89% (27 of 113 cycles) and according to the number of patients was 61.36% (27 of 44 patients). In the 2.5, 5, and 7.5 groups, follicles occurred in 22.9%, 42.1%, and 85.18% of cycles, and pregnancy rate was 14.58%, 28.94% and, 33.33%, respectively. *Conclusions*. It is better to administer Letrozole at a lower dosage to prevent complications and increase the dose based on sonographic results antral follicular count, anti-Müllerian hormone, LH/FSH, and estradiol.

## 1. Introduction

Letrozole is an effective treatment for anovulatory infertile women. Letrozole appears not to have any adverse effects on the endometrium which is frequently associated with clomiphene citrate during ovulation induction [1, 2]. Many researchers have tried letrozole for ovulation induction in different methods [3–9]. Letrozole induced fewer mature follicles that can decrease multiple-pregnancy rate and risk of ovarian hyperstimulation syndrome. Therefore, the letrozole as the first-line drug of ovulation-induction agents in polycystic ovarian syndrome (PCOS) patients can be acceptable [10]. Segawa et al. have accepted letrozole and clomiphene to have the same effect in pregnancy outcome in PCOS patients although letrozole is not toxic and does not have any significant congenital anomaly associations [11–13]. In this study we have recommended letrozole in serial doses of 2.5, 5, and 7.5 mg in each cycle in clomiphene citrate-resistant infertile women with PCOS. The main interest of this paper is the assessment of efficacy and complication of letrozole in follicular size and number, pregnancy rate, abortion, endometrial thickness, and cumulative pregnancy rates in a clinical trial of clomiphene citrate-resistant patients.

## 2. Materials and Methods

In this prospective intervention, we studied Abolfazle Clinic's outpatients of the Bushehr University of Medical Sciences in Iran who referred between January 1, 2008, and December 30, 2010. The study was approved by the institutional ethics committee of the Bushehr University of Medical Sciences, and all patients were required to provide written informed consent before the study commenced. Inclusion criteria are as follows. The study group consisted of polycystic ovarian syndrom (PCOS) patients diagnosed according to the Rotterdam criteria [1]. We defined clomiphene citrate (Clomid, Iran Hormone, Tehran, Iran) resistance as failure to achieve adequate follicular maturation after consumption of 3 cycles of cc at 150 mg/day, determined by serial estradiol monitoring and sonography [1]. Patients resistant to clomiphene citrate became candidates for letrozole (Femara, Novartis, QC, Canada) consumption at the step-up of the protocol. Patients information was recorded in Table 1. Exclusion criteria were abnormal thyroid function test, hyperprolactinoma, galactorrhea, male-factor infertility, tubal and uterine causes of infertility (hysterosalpingography), abnormal response in progesterone challenge test which implies no endogenous estrogen production, FSH > 10, poor patient compliance or complications with treatment. The required information was gathered through demographic and infertility interviews using an information list. Each patient was followed for 6 sessions. Estradiol levels (E2) were measured using the ELISA method by RAYTO set and DIAPLUS kit before the administration of HCG. Serial sonography (Hoda 4000, Japan) was conducted from the 10th day of menstruation and depended on follicular size (18–24 mm) from which HCG (human chorionic gonadotropin, Pregnyl, Organon, Oss, The Netherlands) was administered. There were 3 steps in which we prescribed letrozole. In all cases, daily administration began on the 3rd day of the menstrual cycle through to the 7th day (totalling 5 days). In the first step we prescribed letrozole at a dose of 2.5 mg (one tablet). Normal follicular size and endometrial thickness were considered 18–24 mm and 6 mm or more, respectively, [1]. If the follicle was deemed not acceptable, the dose of letrozole was increased at the next cycle. At the second and third steps we prescribed letrozole at a dose of 5 mg daily and 7.5 mg/day, respectively, and according to patient's response, repeated the same dose. In the current study we tested the hypothesis that prescribing letrozole as an ovulation induction agent in infertile women would increase pregnancy rate, ovulation, and follicle number. The primary outcome measure was normal follicular size, and the secondary outcome measure was the clinical pregnancy. Clinical pregnancy was considered as the presence of a gestational sac with fetal heart activity. Letrozole tablets were prescribed by an experienced nurse who thoroughly explained the method of use to the patients. Sonography was done by an experienced radiologist. Statistical analysis was performed by the Statistical Package for Social Science version 11.5 for windows (SPSS Inc., Chicago, IL, USA). The data was analyzed by student's *t*-test and chi-squared test for linear trend and comparing proportions. A *P* value of <0.05 was considered to be statistically significant.

## 3. Results

**Figure 1:**
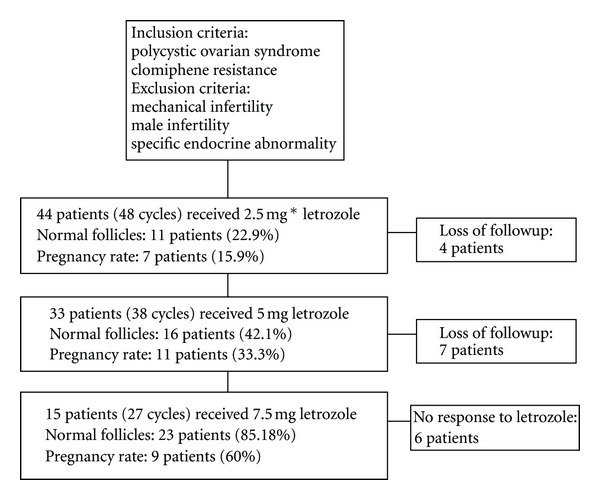
Flow chart of patient's treatment. *mg (milligram).

Demographic information was described in Table 1. These 44 patients had received Clomid in 3 sessions (150 mg), and their sonography results did not show any dominant follicle in serial sonography and were known as clomiphene citrate resistant. According to the flow chart (Figure 1), treatment was started. Number of follicles was summarized in Table 2. There was a significant linear relationship between letrozole dosage and follicular number (*χ* ^2^ for linear trend: 25.6, *P* < 0.0001), and this trend was significant in each step (*χ* ^2^ for linear trend: 11.48, *P* = 0.005) (Table 2). Complications were presented in Table 3. There was a significant linear trend between Letrozole dosage and its complications (*χ* ^2^ for linear trend: 6.38, *P* < 0.011) (Table 3). Characteristic of letrozole cycle was showed in Table 4.

## 4. Discussion

In this study clomiphene citrate-resistant patient had different response to letrozole in a way that 44.24% of the cycles had normal follicles and 23.89% of them resulted in pregnancy. Increasing the dosage can improve the chance of ovulation and pregnancy. Only 7 (15.9%) patients with 2.5 mg letrozole daily became pregnant while 11 patients were pregnant by increasing the dosage to 5 mg, and, among those who did not respond to these dosages, 9 patients became pregnant by increasing it to 7.5 mg. About 61.36% of the patients became pregnant with letrozole although letrozole is more expensive than clomid but cheaper than gonadotropins and so is more cost effective. The metformin-clomiphene citrate combination was seen to increase the ovulatory and pregnancy rate when compared with CC alone. Metformin increased the ovulatory rate in clomiphene citrate failures, also implying increased sensitivity to clomiphene citrate [14]. Akbary-Asbagh et al. and Begum et al. suggest letrozole as an effective treatment for clomiphene citrate-resistant (PCOS) patients [15, 16]. Two of 113 cycles resulted in twins; increasing the dosage improved the chance of two follicles in one cycle. In the first step, like Akbary-Asbagh et al. and Begum et al. study [15, 16], all the cycles had one follicle, but 31.25% and 30.43% of cycles had two follicles in 5 mg and 7.5 mg Letrozole regimen, respectively, even one patient had 3 follicles in a cycle. In Mitwally et al.'s study, for all PCO patients with a serial increase of letrozole dosage (set-up protocol) the same results were confirmed which may be due to the long-term inhibition of estrogen levels (E2) [17]. When a patient responds to clomiphene citrate, the E2 will increase considerably which will be greater than when she receives Letrozole [2, 4]. Because of clomiphene citrate resistance, E2 levels do not increase by clomiphene citrate consumption, and beside ovulation its level will be less than the acceptable level in letrozole groups [6]. Basal testosterone level is a good marker for pregnancy outcome and quantity of dominant follicles on HCG day in women with reduced ovarian reserve but not in women with normal range of serum FSH [18]. Letrozole is an effective ovulation induction drug in elevated-BMI women [19]. We had not any correlation between BMI, basal testosterone, LH/FSH, and number of mature follicle because according to inclusion criteria all of the patients had normal testosterone level, BMI, and LH/FSH ratio. The chance of AUB increased from 2.08% with 2.5 mg letrozole daily to 2.63% with 5 mg daily and 14.8% in the 7.5 mg regimen. No change was found in endometrial thickness according to the Cortines study [6]. So AUB could be due to a decrease in estrogen levels. Given these findings, it is required that more investigations be conducted with estrogen drugs like conjugated estrogens which can prevent AUB in cases with additional letrozole dosage. Letrozole can block estrogen (E) production, consequential in decreased negative feedback of E on pituitary for FSH secretion. Bentov et al., prove that a ratio of cycle day 7 to cycle day 3 postletrozole FSH level >1.5 is related with poor ovarian response. Letrozole challenge test can be a prediction of ovarian response [20]. We suggest that higher pregnancy rates during letrozole treatment can be achieved if antral follicular count, anti-Müllerian hormone, LH/FSH, and estradiol are checked and good patient selection is done [1]. The good pregnancy results and low multiple gestation rate of 2.5 mg letrozole for induction of ovulation is hopeful for letrozole user as a first-line drug [12]. The chances of multigestational pregnancy increases by increasing the number of follicles in a cycle which implies an appropriate response to letrozole. Cumulative pregnancy rates were considerable in clomiphene citrate resistant patients, but the pregnancy rate was not significantly different between the 2.5, 5, and 7.5 mg regimens while complications (multigestational pregnancy risk, irregular bleeding, and ovarian cyst) increased by dosage. Delivery rate was 25 live birth/cycles (22.12%) due to 2 abortions during the study.

## 5. Conclusion

Finally it is concluded that in Clomid-resistant patients, it is better to start letrozole with the lower dosage to prevent more complications and increase dosage based on the sonographic results, antral follicular count, anti-Müllerian hormone, LH/FSH, and estradiol. 

## Figures and Tables

**Table 1:** Patient and cycle characteristics.

Characteristics	Mean ± SD	Range
Age (years)	26.67 ± 5.14	18–39
BMI (kg/m^2^)	25.8 ± 2.4	19.3–29.85
Duration of infertility (years)	3.5 ± 0.82	2–8
Basal FSH (mIU/mL)	5.95 ± 1.48	<10
Basal LH (mIU/mL)	7.12 ± 2.2	<12
Basal Esteradiol (pg/mL)	45.98 ± 21.33	25–75
Basal TSH (*μ*IU/mL).	2.18 ± 1.4	0.88–4
Basal prolactin (ng/mL)	20.14 ± 2.89	<24
DHEAS (Ug/dL)	220 ± 130	35–430
Testosterone (ng/mL)	0.48 ± 0.3	0.1–1.8

**Table 2:** Number of follicles in step-up protocol.

Dosage	One follicle	Multifollicles
Letrozole 2.5 mg	10 (83.33%)	2 (16.66%)
Letrozole 5 mg	11 (68.75%)	5 (31.25%)
Letrozole 7.5 mg	15 (65.21%)	8 (34.78%)

*χ* ^2^ for linear trend: 25.6, *P* < 0.0001.

**Table 3:** Complication of letrozole in step-up protocol.

Dosage	AUB	Ovarian cyct
Letrozole 2.5 mg	2.08%	—
Letrozole 5 mg	2.63%	2.63%
Letrozole 7.5 mg	14.61%	3.7%

*χ* ^2^ for linear trend: 6.38, *P* < 0.011.

**Table 4:** characteristics of letrozole cycles.

Variable	Letrozole groups Mean ± SD	Letrozole total cycle
Follicular development (mm)	20.3 ± 1.89	113
Serum E2 one day of HCG (pg/mL)	120 ± 15.5	113
Pregnancy/cycles	27 (23.89%)	113
Pregnancy/total patients	27 (61.36%)	113
